# Dr. James Bissett Murdoch

**Published:** 1896-12

**Authors:** 


Dr. James Bissett Murdoch, of Pittsburg, died at his home, 4232 Fifth avenue, in that city, October 29, 1896, aged 66 years. He was a native of Glasgow, but came to America when a child and settled in the State of New York. He graduated from the College of Physicians and Surgeons in 1854, served in Bellevue Hospital, was appointed surgeon of one of the Atlantic liners and later located at Oswego. He served as surgeon of the Twenty-fourth New York Volunteers during the first two years of the civil war. He went to Pittsburg in 1872, where he resided until his death. He was an active member of the several medical societies in his vicinity, state, county and city, and of the American Association of Railway Surgeons. He was professor of surgery in Western Pennsylvania medical college, and a member of the surgical staff at the hospital. His name is well known in medical literature. He was a ready debater and so became prominent in medical society discussions. Dr. Murdoch was twice married, and his widow, two sons and two daughters survive him.

